# Supplementary material for: Restoring bone marrow niche function rejuvenates aged hematopoietic stem cells by reactivating the DNA Damage Response
Source: Nat Commun. 2023 Apr 10;14:2018. doi: 10.1038/s41467-023-37783-4 (PMC10086043; doi:10.1038/s41467-023-37783-4)
Supplement: Supplementary file 1 — Supplementary Information [file 41467_2023_37783_MOESM1_ESM.pdf]

Supplementary Information

Restoring bone marrow niche function rejuvenates aged hematopoietic stem cells by reactivating the DNA Damage Response.

Ramalingam et al.

Supplementary Information

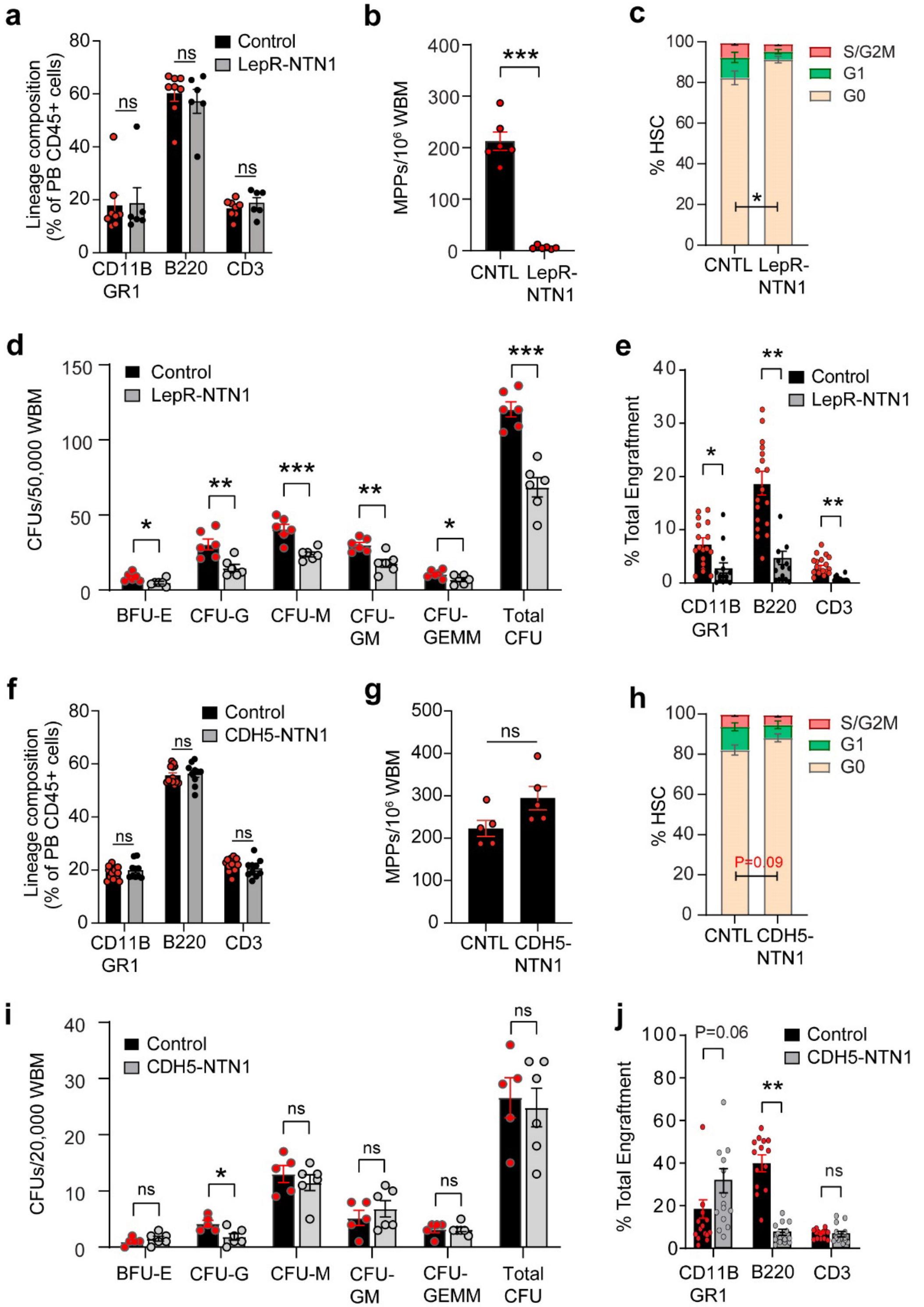

Supplementary Figure 1. BM niche derived NTN1 regulates HSC activity. **(a)** Peripheral blood (PB) analysis demonstrating unaltered lineage composition in young (5-6 month old) *LepR-NTN1* mice, as compared to littermate controls (Controls N=9 mice; *LepR-NTN1* N=7 mice). **(b)** BM analysis of *LepR-NTN1* mice demonstrating a decrease in phenotypic BM MPPs

(N=6 mice/group). **(c)** Cell-cycle analysis (Ki67/Hoechst) demonstrating an increase in quiescence (%G0) in HSCs derived from *LepR-NTN1* mice (N=5 mice/group). **(d)** Colony Forming Unit (CFU) assays demonstrating a decline in BM hematopoietic progenitor activity in *LepR-NTN1* mice, as compared to their littermate controls (N=6 mice/group). **(e)** Absolute engraftment levels of long term engrafted CD45.2+ myeloid, B and T cells following HSC transplantation (Figure 3e). N=6 donors/group; N=18 Recipients for Control donors, N=14 Recipients for *LepR-NTN1* donors. **(f)** Peripheral blood analysis demonstrating unaltered lineage composition in young *CDH5-NTN1* (5-6 month old) mice, as compared to littermate controls (N=10 mice/group). **(g)** BM analysis of *CDH5-NTN1* mice demonstrating no changes in phenotypic BM MPPs (N=5 mice/group). **(h)** Cell-cycle analysis (Ki67/Hoechst) demonstrating a modest increase in quiescence (%G0) in HSCs derived from *CDH5-NTN1* mice (N=5 mice/group). **(i)** CFU assays demonstrating no gross changes in overall progenitor activity in *CDH5-NTN1* mice (N=5 mice/group). **(j)** Absolute engraftment levels of long term engrafted CD45.2+ myeloid, B and T cells following HSC transplantation (Figure 3k). N=6 donors/group; N=14 recipients/group. Data is presented as the mean ± standard error of the mean (SEM). Statistical significance determined using two-tailed unpaired t-test. * $P<0.05$; ** $P<0.01$; *** $P<0.001$. ns denotes statistically not significant.

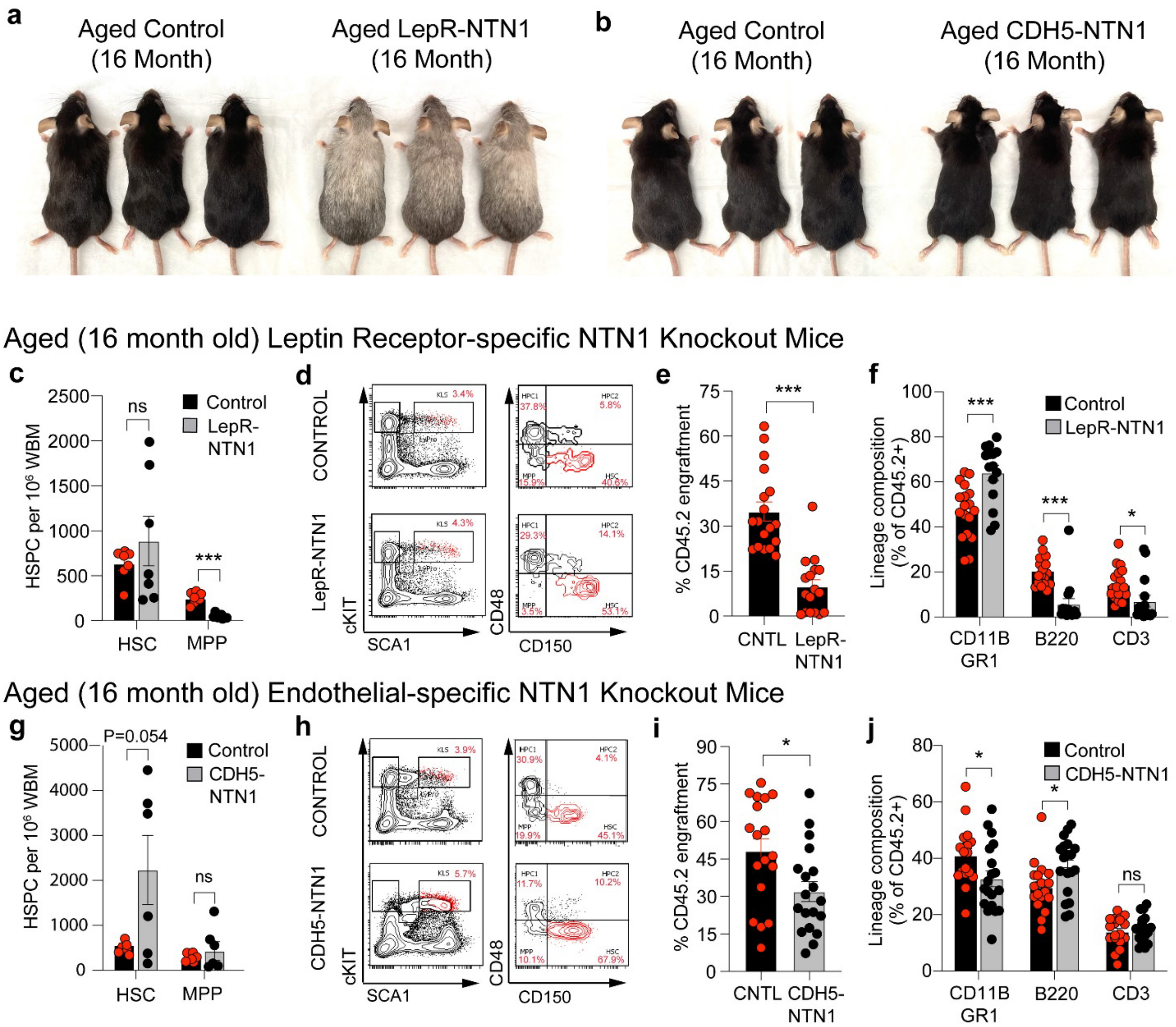

Supplementary Figure 2. Niche derived NTN1 preserves HSC fitness during aging. (a, b) Representative images demonstrating hair greying in aged *Lepr-NTN1* mice **(a)**, but not in aged *CDH5-NTN1* mice **(b)**. **(c, d)** BM analysis of aged *LepR-NTN1* mice demonstrating a decrease in MPPs, as compared to littermate controls (N=7 mice/group). **(e-f)** Competitive HSC transplantation assays (1,000 CD45.2+ donor HSCs with 10^6 CD45.1 WBM competitor/recipient) demonstrating diminished long-term (>6 months) engraftment **(e)**, and a myeloid-biased reconstitution **(f)**, in HSCs derived from aged *LepR-NTN1* mice as compared to littermate controls (N=6 donors/group, N=18 Recipients for Control donors, N=17 Recipients for *LepR-NTN1* donors). **(g, h)** BM analysis of aged *CDH5-NTN1* mice demonstrating an increase in phenotypic HSCs **(g)**, as compared to littermate controls (N=6 mice/group). **(i, j)** Competitive HSC transplantation assays demonstrating diminished long-term engraftment **(i)**, and altered lineage reconstitution **(j)**, in HSCs derived from aged *CDH5-NTN1* mice as compared to littermate controls (N=6 donors/group, N=18 Recipients for Control donors, N=19 Recipients for *CDH5-NTN1* donors). Note the increased variance in HSC frequency in both aged *LepR-NTN1* mice and *CDH5-NTN1* mice indicative of a stressed hematopoietic system. Data is presented as the mean ± standard error of the mean (SEM). Statistical significance determined using two-tailed unpaired t-test. * P<0.05; ** P<0.01; *** P<0.001. ns denotes statistically not significant.

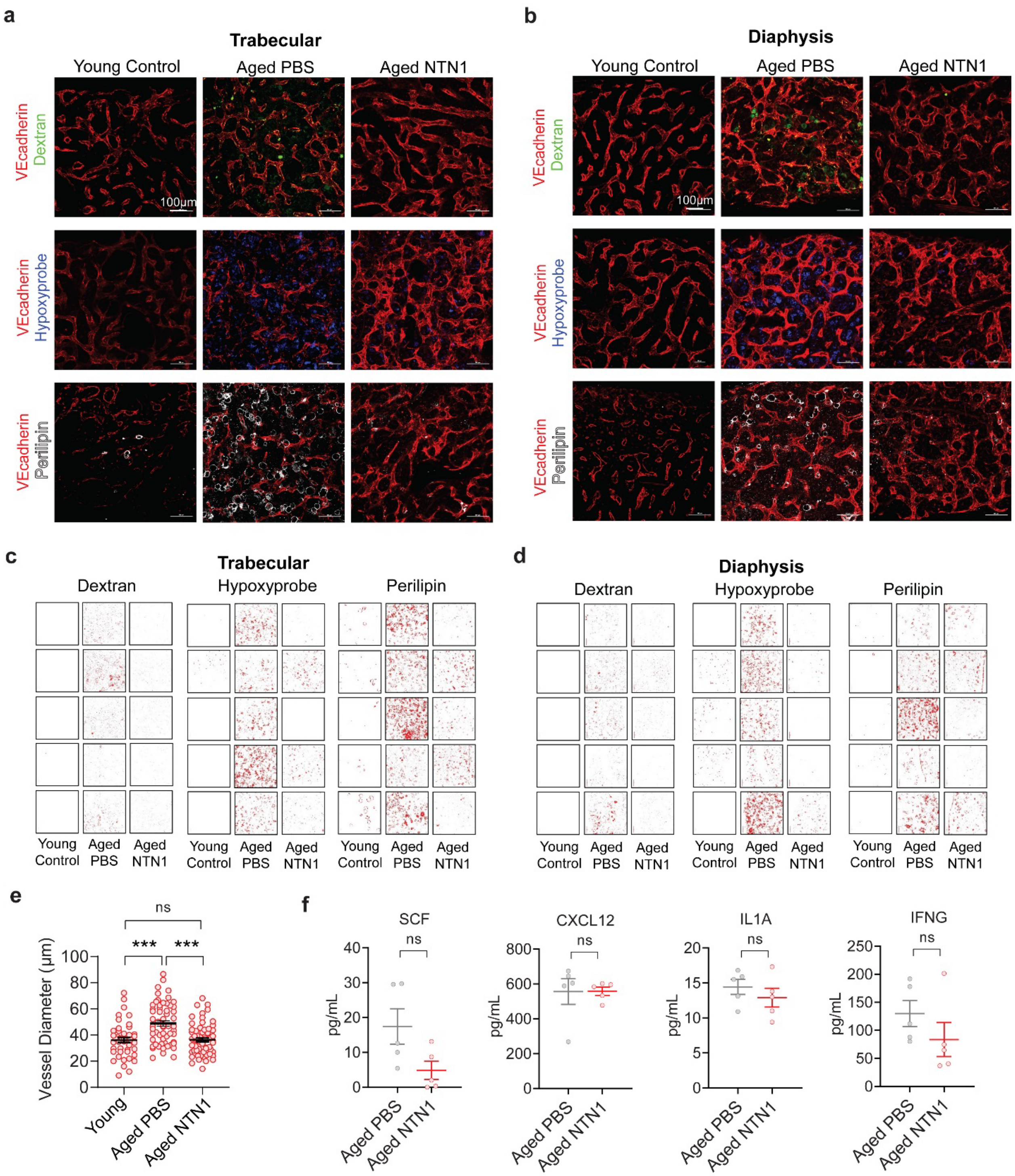

Supplementary Figure 3. NTN1 revitalizes the aged BM niche. (a, b) Representative IF images of BM femoral sections for analysis of BM niche function following NTN1 treatment of aged mice by determining vascular leakiness (10 kDa dextran extravasation), vascular perfusion (Hypoxyprobe staining), and BM adiposity (PLIN1+ adipocytes/high power field) in both trabecular **(a)**, and diaphyseal **(b)** regions of BM (N=5 mice/group). **(c, d)** Thresholded IF images (Image J) of trabecular **(c)** and diaphyseal **(d)** regions of femurs from individual mice

utilized for quantification in Figures 7b & 7c. **(e)** Quantification of vessel diameter within the BM of aged mice following NTN1 supplementation (N=5 mice/group). Statistical significance determined using One-way ANOVA analysis with a Tukey's Correction. **(f)** Quantification of cytokines (ELISA) in the BM supernatant of aged mice treated with PBS/NTN1 (N=5 mice/group). Statistical significance determined using two-tailed unpaired t-test. Data is presented as the mean ± standard error of the mean (SEM). Statistical significance is indicated as *** ($p < 0.001$), and n.s. (not significant).

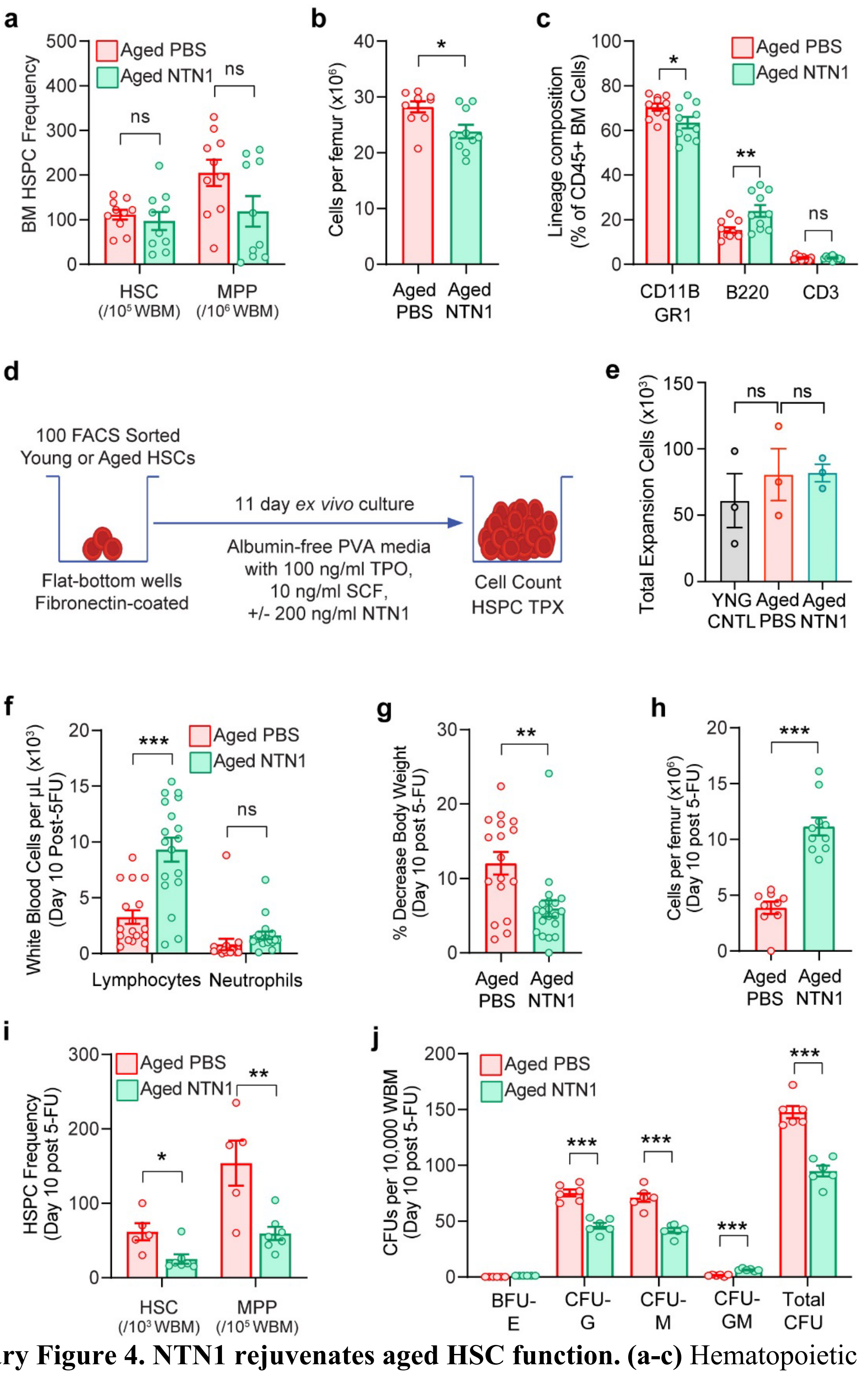

Supplementary Figure 4. NTN1 rejuvenates aged HSC function. (a-c) Hematopoietic analysis of aged mice at steady state following 10 injections of PBS/NTN1. **(a)** Phenotypic HSPC frequency (N=10 mice/group). **(b)** Quantification of BM cellularity (N=10 mice/group). **(c)** Evaluation of lineage composition by flow cytometry (N=10 mice/group). **(d)** Experimental scheme to assess the direct effects of NTN1 treatment on aged HSC function. **(e)** Average HSPC expansion in each well after 11 days of culture *ex vivo*. Each dot represents average number of

cells across 3-5 replicate cultures for each HSC donor (N=3 donors/group; N=13-15 expansion wells/group). **(f-j)** Analysis at day 10 post single-dose 5-FU demonstrating higher levels of peripheral blood lymphocytes and neutrophils **(f)** (PBS N=17 mice, NTN1 N=18 mice), preservation of body weight **(g)** (PBS N=17 mice, NTN1 N=20 mice), improved BM cellularity **(h)** (PBS N=9 mice, NTN1 N=10 mice), a decrease in HSPC frequency **(i)** (PBS N=5 mice, NTN1 N=7 mice), and a reduction in BM progenitor activity **(j)** (N=6 mice/group) in aged mice treated with NTN1, as compared to PBS controls. Data is presented as the mean ± standard error of the mean (SEM). Statistical significance determined using two-tailed unpaired t-test (for pairwise comparisons), and One-Way ANOVA with Tukey's correction for multiple comparisons. * P<0.05; ** P<0.01; *** P<0.001. ns denotes statistically not significant.

a

REACTOME Pathways

Upregulated in Young and Aged NTN1 HSCs (P < 0.05, FDR < 0.1)	NES (Young)	NES (Aged NTN1)
CELL CYCLE CHECKPOINTS	2.98	1.71
G2M CHECKPOINTS	2.64	1.58
ACTIVATION OF ATR IN RESPONSE TO REPLICATION STRESS	2.63	2.11
BASE EXCISION REPAIR	2.44	1.71
RESOLUTION OF ABASIC SITES_AP SITES	2.25	1.71
MISMATCH REPAIR	2.17	1.59

b

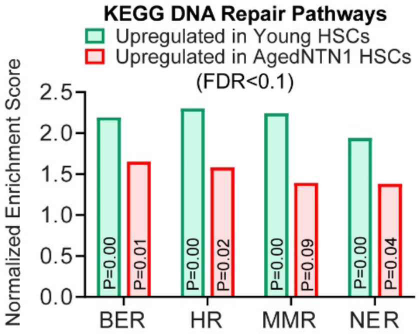

c

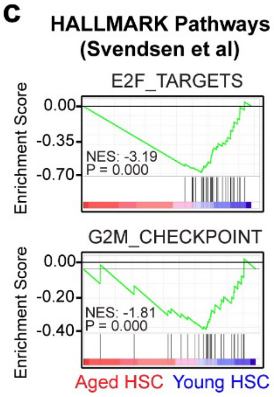

d

REACTOME Pathways (Svendsen et al)

Downregulated in Aged HSCs (P < 0.05, FDR < 0.1)	NES
CELL_CYCLE_CHECKPOINTS	-2.71
DNA_REPLICATION	-2.62
CELL_CYCLE_MITOTIC	-2.31
CELL_CYCLE	-2.29
S_PHASE	-2.29
MITOTIC_G1_PHASE_AND_G1_S_TRANSITION	-2.06
DNA_REPAIR	-1.59

e

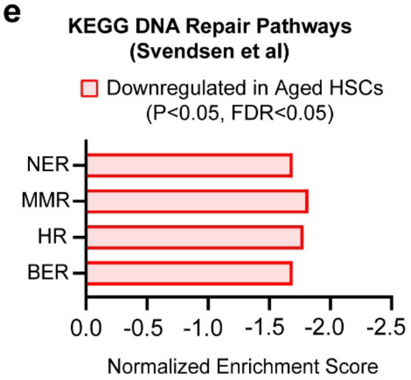

f

DDR Genes Downregulated in Aged HSCs (Svendsen et al)

ASF1B	CENPA	DTYMK	GINS2	LIG1	MSH6	POLA2	RAD54L	TACC3
BLM	CIT	DYNLL1	GMNN	LMNB1	MTHFD2	POLD1	RFC2	TCF19
CCND2	DNA2	ERCC6L	KIF22	MAD1L1	NUP133	POLE	RMI2	TIMELESS
CDC6	DNMT1	EXO1	KIF23	MCM2	NUP35	PRIM1	RPA2	UNG
CDCA3	DSCC1	EZH2	KIF2C	MCM5	NUSAP1	RAD51	RRM2	USP1
CDCA5	DTL	FEN1	KMT5A	MCM7	ORC1	RAD52	SYNCRIP	XRCC5

Supplementary Figure 5. DDR downregulation is a conserved feature of HSC aging. (a) GSEA with the REACTOME database showing an upregulation of cell-cycle checkpoints and DNA repair pathways in young HSCs and aged NTN1 HSCs, when compared with aged PBS HSCs. **(b)** DNA repair pathways upregulated in young HSCs and aged NTN1 HSCs, when compared with aged PBS HSCs. **(c-f)** GSEA of genes identified to demonstrate consistent alterations during HSC aging, in a meta-analysis of published HSC aging transcriptomes. **(c)** GSEA enrichment plots demonstrating a downregulation of E2F_TARGETS and G2M_CHECKPOINT pathways in aged HSCs, as compared to young HSCs. **(d, e)** GSEA with the REACTOME **(d)**, and KEGG pathways **(e)**, demonstrating downregulation of DNA repair pathways in aged HSCs. **(f)** DDR genes that are downregulated in aged HSCs, identified in the comprehensive HSC aging meta-analysis dataset. Normalized enrichment scores (NES) and p-values adjusted for multiple comparisons were obtained utilizing default parameters of GSEA analysis.

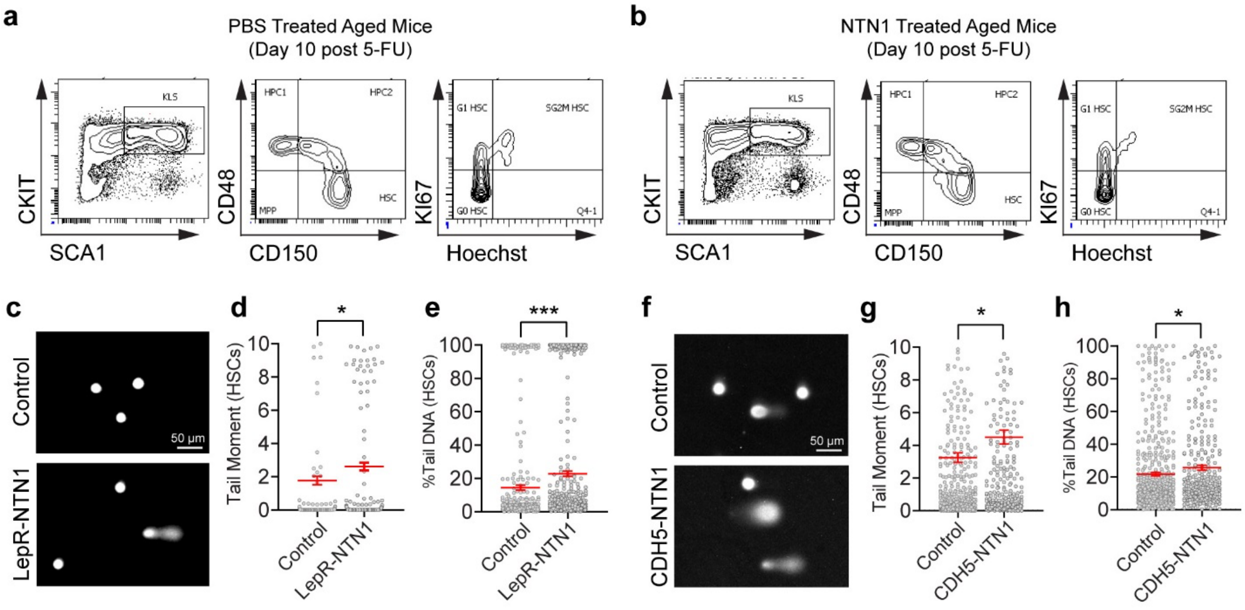

Supplementary Figure 6. Loss of niche-derived NTN1 induces DNA damage within HSCs. **(a, b)** Representative flow cytometry contour plots for assessment of HSC cell cycle status following 5-FU administration and treatment with PBS or NTN1. **(c)** Representative IF images of alkaline comet assays in HSCs derived from *LepR-NTN1* mice. **(d, e)** Alkaline comet analysis demonstrating an increase in average Tail-Moment and % Tail DNA in HSCs derived from *LepR-NTN1* mice (N=3 mice/group). **(f)** Representative IF images of alkaline comet assays in HSCs derived from *CDH5-NTN1* mice. **(g, h)** Alkaline comet analysis demonstrating an increase in average Tail-Moment and % Tail DNA in HSCs derived from *CDH5-NTN1* mice (N=3 mice/group). Note that deletion of NTN1 within either MSCs or BMECs results in increased DNA damage within HSCs. Data is presented as the mean ± standard error of the mean (SEM). Statistical significance determined using two-tailed unpaired t-test. * P<0.05; ** P<0.01; *** P<0.001.

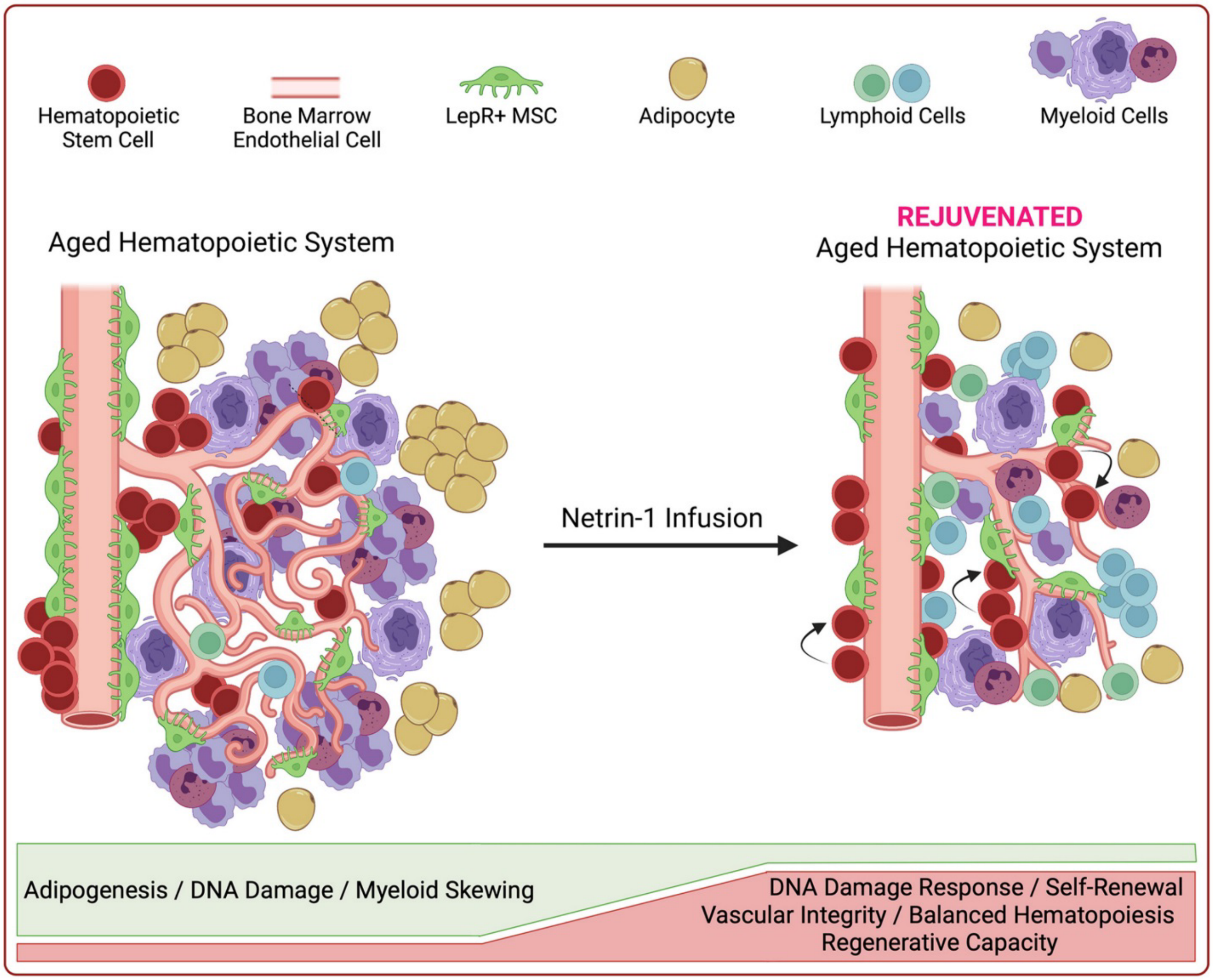

Supplementary Figure 7. Schematic describing the role of NTN1 in preserving DDR capacity and functionality of LepR+ MSCs, ECs and HSCs within the BM niche. NTN1 preserves vascular integrity, suppresses MSC adipocyte differentiation and promotes HSC self-renewal in young mice, by maintaining an active DDR (Right Panel). Aging is associated with a decline in niche-derived NTN1 and dampened DDR, contributing towards defects in vascular niche integrity, MSC activity and HSC fitness (Left Panel). Exogenous supplementation with NTN1 reactivates the dampened DDR within BMECs and HSCs, which is sufficient to reverse the hallmark defects characterizing an aged hematopoietic system. Figure generated using BioRender.
