# Supplementary material for: Restoring bone marrow niche function rejuvenates aged hematopoietic stem cells by reactivating the DNA Damage Response
Source: Nat Commun. 2023 Apr 10;14:2018. doi: 10.1038/s41467-023-37783-4 (PMC10086043; doi:10.1038/s41467-023-37783-4)
Supplement: Supplementary file 3 — Description of Additional Supplementary Files [file 41467_2023_37783_MOESM3_ESM.pdf]

Description of Additional Supplementary Files:

Supplementary Data 1: RNA-Seq FPKM of LepR+ MSCs and BMECs in mice with conditional deletion of NTN1 in MSCs (LepR-NTN1 mice) and BMECs (CDH5-NTN1).

Supplementary Data 2: RNA-Seq Pathway Analysis of LepR+ MSCs in mice with conditional deletion of NTN1 in MSCs (LepR-NTN1 mice) and BMECs (CDH5-NTN1).

Supplementary Data 3: RNA-Seq Pathway Analysis of BMECs in mice with conditional deletion of NTN1 in MSCs (LepR-NTN1 mice) and BMECs (CDH5-NTN1).

Supplementary Data 4: RNA-Seq FPKM of LepR+ MSCs derived from young (3 month old) and aged (18 month old) C57BL6 mice.

Supplementary Data 5: RNA-Seq Pathway Analysis of LepR+ MSCs derived from young (3 month old) and aged (18 month old) C57BL6 mice.

Supplementary Data 6: RNA-Seq FPKM of BMECs derived from young (3 month old) and aged (18 month old) C57BL6 mice.

Supplementary Data 7: RNA-Seq Pathway Analysis of BMECs derived from young (3 month old) and aged (18 month old) C57BL6 mice.

Supplementary Data 8: RNA-Seq FPKM of LepR+ MSCs and BMECs derived from aged (18 month old) C57BL6 mice treated with PBS or recombinant Netrin-1.

Supplementary Data 9: RNA-Seq Pathway Analysis of LepR+ MSCs and BMECs derived from aged (18 month old) C57BL6 mice treated with PBS or recombinant Netrin-1.

Supplementary Data 10: RNA-Seq FPKM of HSCs derived from young (3 month old) and PBS/NTN1 treated aged (18 month old) C57BL6 mice.

Supplementary Data 11: RNA-Seq Pathway Analysis of HSCs derived from young (3 month old) and PBS/NTN1 treated aged (18 month old) C57BL6 mice.

Supplementary Data 12: RNA-Seq Pathway Analysis of a published meta-analysis of HSC Aging transcriptomic datasets (Svendsen et al).

Supplementary Data 13: Antibodies and Reagents
