# Supplementary material for: Restoring bone marrow niche function rejuvenates aged hematopoietic stem cells by reactivating the DNA Damage Response
Source: Nat Commun. 2023 Apr 10;14:2018. doi: 10.1038/s41467-023-37783-4 (PMC10086043; doi:10.1038/s41467-023-37783-4)
Supplement: Supplementary file 17 — Reporting Summary [file 41467_2023_37783_MOESM17_ESM.pdf]

Corresponding author(s): Jason M. Butler
Last updated by author(s): Mar 20, 2023

Reporting Summary

Nature Portfolio wishes to improve the reproducibility of the work that we publish. This form provides structure for consistency and transparency in reporting. For further information on Nature Portfolio policies, see our Editorial Policies and the Editorial Policy Checklist.

Statistics

For all statistical analyses, confirm that the following items are present in the figure legend, table legend, main text, or Methods section.

n/a	Confirmed	
☐	☒	The exact sample size (*n*) for each experimental group/condition, given as a discrete number and unit of measurement
☐	☒	A statement on whether measurements were taken from distinct samples or whether the same sample was measured repeatedly
☐	☒	The statistical test(s) used AND whether they are one- or two-sided<br>*Only common tests should be described solely by name; describe more complex techniques in the Methods section.*
☒	☐	A description of all covariates tested
☐	☒	A description of any assumptions or corrections, such as tests of normality and adjustment for multiple comparisons
☐	☒	A full description of the statistical parameters including central tendency (e.g. means) or other basic estimates (e.g. regression coefficient) AND variation (e.g. standard deviation) or associated estimates of uncertainty (e.g. confidence intervals)
☐	☒	For null hypothesis testing, the test statistic (e.g. *F*, *t*, *r*) with confidence intervals, effect sizes, degrees of freedom and *P* value noted<br>*Give P values as exact values whenever suitable.*
☒	☐	For Bayesian analysis, information on the choice of priors and Markov chain Monte Carlo settings
☒	☐	For hierarchical and complex designs, identification of the appropriate level for tests and full reporting of outcomes
☒	☐	Estimates of effect sizes (e.g. Cohen's *d*, Pearson's *r*), indicating how they were calculated

Our web collection on statistics for biologists contains articles on many of the points above.

Software and code

Policy information about availability of computer code

Data collection	No custom code utilized for data collection.
Data analysis	1. iDEP (http://bioinformatics.sdstate.edu/idep94/)<br>2. GraphPad Prism<br>3. Image J<br>4. Extreme Limiting Dilution Analysis (ELDA) software (http://bioinf.wehi.edu.au/software/elda/).

For manuscripts utilizing custom algorithms or software that are central to the research but not yet described in published literature, software must be made available to editors and reviewers. We strongly encourage code deposition in a community repository (e.g. GitHub). See the Nature Portfolio guidelines for submitting code & software for further information.

Data

Policy information about availability of data

All manuscripts must include a data availability statement. This statement should provide the following information, where applicable:

- Accession codes, unique identifiers, or web links for publicly available datasets
- A description of any restrictions on data availability
- For clinical datasets or third party data, please ensure that the statement adheres to our policy

The authors declare that data supporting the findings of this study are available within the manuscript and its supplementary information files. Source data for all Figures are provided with the paper.

Human research participants

Policy information about studies involving human research participants and Sex and Gender in Research.

Reporting on sex and gender	This study does not involve human participants/samples.
Population characteristics	*Describe the covariate-relevant population characteristics of the human research participants (e.g. age, genotypic information, past and current diagnosis and treatment categories). If you filled out the behavioural & social sciences study design questions and have nothing to add here, write "See above."*
Recruitment	*Describe how participants were recruited. Outline any potential self-selection bias or other biases that may be present and how these are likely to impact results.*
Ethics oversight	*Identify the organization(s) that approved the study protocol.*

Note that full information on the approval of the study protocol must also be provided in the manuscript.

Field-specific reporting

Please select the one below that is the best fit for your research. If you are not sure, read the appropriate sections before making your selection.

☒ Life sciences ☐ Behavioural & social sciences ☐ Ecological, evolutionary & environmental sciences

For a reference copy of the document with all sections, see nature.com/documents/nr-reporting-summary-flat.pdf

Life sciences study design

All studies must disclose on these points even when the disclosure is negative.

Sample size	Sample sizes for phenotypic and functional analysis of mouse hematopoietic parameters were determined based on prior estimates of variance and effect sizes observed in previous experiments. Number of animals needed were calculated based on the ability to detect a two-fold change in the Mean with 80% power, with the threshold for significance (α) set at 0.05.
Data exclusions	Animals which developed illnesses (tumors, severe dermatitis) were excluded from the study.
Replication	All hematopoietic functional analyses including transplantations were performed at least twice to ensure reproducibility.
Randomization	Aged animals were randomly assigned to PBS and Netrin-1 treatment groups. All aged animals within individual experiments were matched for age and gender.
Blinding	Blinding was not used.

Reporting for specific materials, systems and methods

We require information from authors about some types of materials, experimental systems and methods used in many studies. Here, indicate whether each material, system or method listed is relevant to your study. If you are not sure if a list item applies to your research, read the appropriate section before selecting a response.

Materials & experimental systems

n/a	Involved in the study	
☐	☒	Antibodies
☐	☒	Eukaryotic cell lines
☒	☐	Palaeontology and archaeology
☐	☒	Animals and other organisms
☒	☐	Clinical data
☒	☐	Dual use research of concern

Methods

n/a	Involved in the study	
☒	☐	ChIP-seq
☐	☒	Flow cytometry
☒	☐	MRI-based neuroimaging

Antibodies

Antibodies used	All antibodies used in the study are Listed in Supplemental Data 13
Validation	No new antibodies were generated in this study. All antibodies are commercially available with detailed descriptions available in the manufacturer's websites (Supplemental Data 13).

Eukaryotic cell lines

Policy information about cell lines and Sex and Gender in Research

Cell line source(s)	Mouse bone marrow-derived endothelial cells and mesenchymal stromal cells.
Authentication	Endothelial and mesenchymal stromal identity validated by Flow cytometry, as described in the Methods.
Mycoplasma contamination	Not determined.
Commonly misidentified lines (See ICLAC register)	Not applicable.

Animals and other research organisms

Policy information about studies involving animals; ARRIVE guidelines recommended for reporting animal research, and Sex and Gender in Research

Laboratory animals	C57BL/6 (CD45.2, JAX Stock No: 000664) mice were purchased from the National Institutes on Aging, and the Jackson Laboratory (Bar Harbor, ME). B6.129(Cg)-Leprtm2(cre)Rck/J (JAX Stock No. 008320) mice, B6.129(SJL)-NTN1tm1.1Tek/J (JAX Stock No. 028038) mice and B6.SJL-Ptprca Pepcb/BoyJ (CD45.1; JAX stock No. 002014) mice were purchased from the Jackson Laboratory. Cdh5(PAC)-creERT2 mice were obtained from Ralf H. Adams at The Max Planck Institute for Molecular Biomedicine.
Wild animals	No wild animals utilized.
Reporting on sex	Both male and female animals were utilized in the study. Gender proportion was maintained across experimental groups within each experiment.
Field-collected samples	Not applicable.
Ethics oversight	All murine experiments were conducted in accordance with the Association for Assessment and Accreditation of Laboratory Animal Care, Intl. (AAALAC) and National Institutes of Health (NIH) Office of Laboratory Animal Welfare (OLAW) guidelines, and under the approval of the Institutional Animal Care and Use Committee (IACUC) at Hackensack Meridian Health and Center for Discovery and Innovation, and the University of Florida.

Note that full information on the approval of the study protocol must also be provided in the manuscript.

Flow Cytometry

Plots

Confirm that:

☐ The axis labels state the marker and fluorochrome used (e.g. CD4-FITC).

☒ The axis scales are clearly visible. Include numbers along axes only for bottom left plot of group (a 'group' is an analysis of identical markers).

☒ All plots are contour plots with outliers or pseudocolor plots.

☒ A numerical value for number of cells or percentage (with statistics) is provided.

Methodology

Sample preparation	Information included in the Methods section.
Instrument	Information included in the Methods section.
Software	Information included in the Methods section.
Cell population abundance	Information included in the Methods section and the Manuscript Figures.
Gating strategy	Information included in the Methods section as well as the Figures and Supplemental Figures for Gating strategies for cell populations utilized in the study.

[x] Tick this box to confirm that a figure exemplifying the gating strategy is provided in the Supplementary Information.
